# *In Utero* and Childhood Polybrominated Diphenyl Ether Exposures and Body Mass at Age 7 Years: The CHAMACOS Study

**DOI:** 10.1289/ehp.1408417

**Published:** 2015-02-27

**Authors:** Ayca Erkin-Cakmak, Kim G. Harley, Jonathan Chevrier, Asa Bradman, Katherine Kogut, Karen Huen, Brenda Eskenazi

**Affiliations:** 1 Center for Environmental Research and Children’s Health (CERCH), School of Public Health, University of California, Berkeley, Berkeley, California, USA; 2 Department of Epidemiology, Biostatistics and Occupational Health, McGill University Faculty of Medicine, Montréal, Québec, Canada

## Abstract

**Background:**

Polybrominated diphenyl ethers (PBDEs) are lipophilic flame retardants that bioaccumulate in humans. Child serum PBDE concentrations in California are among the highest worldwide. PBDEs may be associated with obesity by disrupting endocrine systems.

**Objective:**

In this study, we examined whether pre- and postnatal exposure to the components of pentaBDE mixture was associated with childhood obesity in a population of Latino children participating in a longitudinal birth cohort study in the Salinas Valley, California.

**Methods:**

We measured PBDEs in serum collected from 224 mothers during pregnancy and their children at 7 years of age, and examined associations with body mass index (BMI) at age 7 years.

**Results:**

Maternal PBDE serum levels during pregnancy were associated with higher BMI *z*-scores in boys (BMI *z*-score β_adjusted_ = 0.26; 95% CI: –0.19, 0.72) but lower scores in girls (BMI *z*-score β_adjusted_ = –0.41; 95% CI: –0.87, –0.05) at 7 years of age (*p*_interaction_ = 0.04). In addition, child’s serum BDE-153 concentration (log_10_), but not other pentaBDE congeners, demonstrated inverse associations with BMI at age 7 years (BMI *z*-score β_adjusted_ = –1.15; 95% CI: –1.53, –0.77), but there was no interaction by sex.

**Conclusions:**

We estimated sex-specific associations with maternal PBDE levels during pregnancy and BMI at 7 years of age, finding positive associations in boys and negative associations in girls. Children’s serum BDE-153 concentrations were inversely associated with BMI at 7 years with no difference by sex. Future studies should examine the longitudinal trends in obesity with PBDE exposure and changes in hormonal environment as children transition through puberty, as well as evaluate the potential for reverse causality.

**Citation:**

Erkin-Cakmak A, Harley KG, Chevrier J, Bradman A, Kogut K, Huen K, Eskenazi B. 2015. *In utero* and childhood polybrominated diphenyl ether exposures and body mass at age 7 years: the CHAMACOS Study. Environ Health Perspect 123:636–642; http://dx.doi.org/10.1289/ehp.1408417

## Introduction

Polybrominated diphenyl ethers (PBDEs) are flame retardants that have been used extensively in consumer products since the 1970s in three technical mixtures (penta-, octa-, and decabromo diphenyl ethers) (Besis and Samara 2012; Hale et al. 2003). PBDEs are lipophilic, accumulate in living organisms, and have an estimated half-life up to 12 years in humans (Geyer et al. 2004; Li et al. 2008; Wong et al. 2013). PBDE serum levels are about 20 times higher in the United States than Europe; Californians have the highest levels, likely due to state furniture flammability standards (Eskenazi et al. 2011; Sjödin et al. 2008; Zota et al. 2008). Although the pentaBDE mixture used in furniture, carpet padding, and infant products has been banned since 2004, pentaBDE congeners continue to be released from older furniture and are commonly found in house dust (Noyes et al. 2011).

PBDEs have been detected in cord blood, placental tissue, and breast milk and are transferred pre- and postnatally from women to their children (Herbstman et al. 2010; Wu et al. 2009). Young children’s hand-to-mouth behavior may contribute to additional exposures via oral and dermal contact with dust (Bradman et al. 2012; Lorber 2008).

Obesity is a growing public health problem worldwide (Booth et al. 2001; Lobstein 2004; Popkin and Doak 1998). Although obesity is primarily attributable to genetic predisposition, high calorie intake, and insufficient physical activity, exposure to endocrine-disrupting chemicals such as PBDEs may also play a role (Janesick and Blumberg 2011a; Legler and Brouwer 2003). Estrogen and androgen receptors in the brain and peripheral organs modulate programming of energy balance and distribution of body fat, and PBDEs are known to disrupt estrogen and androgen signaling (Legler et al. 2011). Although lower brominated PBDEs, including BDEs 28, 47, and 100, exhibit estrogenic activity, higher brominated compounds, such as BDE-153, show antiestrogenic properties (Meerts et al. 2001). PBDEs’ potential effect on obesity may also be caused by the disruption of thyroid hormone homeostasis, which regulates the basal metabolic rate and lipid metabolism. PBDE exposure is associated with reduced thyroxine (T_4_) in animal studies (Hallgren et al. 2001; Kuriyama et al. 2007; Zhou et al. 2002) and increased T_4_ or decreased thyroid-stimulating hormone (TSH) in human studies (Chevrier et al. 2010; Hagmar et al. 2001; Turyk et al. 2008). PBDEs may also have direct effects on lipid metabolism and adipogenesis (Hoppe and Carey 2007). In rodents exposed postnatally, pentaPBDEs cause an increase in *in vitro* isoproterenol-stimulated lipolysis and a decrease in insulin-stimulated glucose oxidation, but no effect on fat pad weight, adipocyte number, or adipocyte size (Hoppe and Carey 2007). PPARγ (peroxisome proliferator-activated receptor gamma) is the master regulator of adipocyte development, and activation of PPARγ can lead to adipogenesis and obesity. Although organotins (such as tributylin) and phthalates target PPARγ (Janesick and Blumberg 2011b), as of yet there is no evidence that PBDEs affect PPARγ activity.

In humans, results are conflicting as to whether postnatal PBDE exposure is related to childhood obesity (Lim et al. 2008; Turyk et al. 2010; Windham et al. 2010). However, no previous study has examined the effects of prenatal exposure. In the present study, we examined the association between pre- and postnatal exposure to pentaBDE congeners and measures of body mass in a population of California children participating in the Center for the Health Assessment of Mothers and Children of Salinas (CHAMACOS) study. Because of the purported effects of PBDEs on endocrine function, we also examined differences by child sex.

## Methods

*Participants and recruitment*. Subjects in this study participated in CHAMACOS, a longitudinal birth cohort study investigating the effects of environmental exposures on the health of pregnant women and their children. Detailed methods are described elsewhere (Eskenazi et al. 2003, 2004). Briefly, pregnant women were enrolled between October 1999 and October 2000 from prenatal clinics serving low-income, Spanish-speaking residents in the Salinas Valley, California. Eligible women were at least 18 years of age, < 20 weeks gestation, qualified for low-income health insurance, spoke English or Spanish, and planned to deliver at a local hospital. Of 601 women initially enrolled, 531 were followed to the live birth of an infant. We excluded twins (*n* = 5), children not followed to 7 years of age (*n* = 172), and children whose mothers did not have adequate serum volumes for PBDE measurements (*n* = 55). We further excluded mother–child pairs who were missing PBDE measurements both during pregnancy and at child’s 7 years of age (*n* = 70), leaving a final sample size of 224. Compared with children in the cohort who were not followed, children included in the present analyses were more likely to be female and less likely to have low birth weight, with mothers who were older and breastfed longer (data not shown). They did not differ according to other sociodemographic characteristics listed in Table 1.

**Table 1:** Prenatal and childhood blood PBDE concentrations (ng/g lipid) by demographic characteristics of the study population.

Characteristic	Maternal serum Σ4PBDE^*a*^		Child serum Σ4PBDE^*a*^	
	*n* (%)	GM (95% CI)	*n* (%)	GM (95% CI)
Overall GM (95% CI)	224 (100)	25.35 (22.42, 28.45)	216 (100)	83.03 (74.80, 92.17)
Maternal characteristics at the time of pregnancy (*n* = 224)
Maternal age (years)
18–24	101 (45.1)	26.1 (21.5, 31.8)	96 (45.5)	78.2 (66.9, 91.4)
25–29	80 (35.7)	22.6 (19.0, 26.7)	77 (36.5)	90.5 (76.5, 107.0)
≥ 30	43 (19.2)	29.3 (21.2, 40.7)	38 (18.0)	81.3 (61.8, 106.8)
Maternal education
Less than high school	101 (45.1)	21.9 (18.6, 25.7)	96 (45.5)	75.1 (65.0, 86.7)
Some high school	75 (33.5)	27.2 (21.5, 34.3)	68 (32.2)	88.3 (72.4, 107.5)
High school grad	48 (21.4)	31.0 (23.4, 41.2)	47 (22.3)	93.4 (73.9, 118.1)
Years of residence in USA
≤ 5	114 (50.9)	21.2 (17.5, 25.6)	110 (52.1)	76.0 (66.6, 86.8)
6–10	53 (23.6)	27.3 (22.7, 33.0)	51 (24.2)	86.4 (67.7, 110.2)
≥ 11	34 (15.2)	28.2 (21.3, 37.4)	29 (13.7)	91.0 (64.2, 128.9)
Entire life	23 (10.3)	44.7 (30.2, 66.3)	21(10.0)	105.5 (79.8,139.6)
Prepregnancy maternal BMI (kg/m^2^)^*b*^
Underweight/normal	80 (35.7)	21.9 (18.1, 26.6)	75 (35.5)	82.3 (68.8, 98.5)
Overweight	90 (40.2)	27.3 (22.6, 33.0)	86 (40.8)	82.6 (70.5, 96.8)
Obese	54 (24.1)	27.8 (20.8, 37.1)	50 (23.7)	84.9 (67.3, 107.2)
Parity
0	77 (34.4)	24.0 (19.0, 30.3)	74 (35.1)	69.1 (58.3, 82.1)
≥ 1	147 (65.6)	26.1 (22.7, 30.1)	137 (64.9)	91.7 (80.5, 104.4)
Breastfeeding (months)
< 6	97 (43.3)	28.1 (23.1, 34.3)	88 (41.7)	82.5 (69.8, 97.5)
6 to < 12	55 (24.6)	25.2 (20.3, 31.4)	53 (25.1)	75.0 (62.6, 89.8)
≥ 12	72 (32.1)	22.1 (17.8, 27.5)	70 (33.2)	90.4 (74.3, 110.0)
Child characteristics at birth (*n* = 219)
Low infant birth weight
No	219 (97.8)	25.5 (22.5, 28.9)	206 (97.6)	82.5 (74.3, 91.7)
Yes	5 (2.2)	20.4 (11.7, 35.6)	5 (2.4)	107.1 (38.4, 298.7)
Sex
Male	99 (44.2)	23.4 (19.2, 28.4)	93 (44.1)	83.0 (71.1, 96.9)
Female	125 (55.8)	27.0 (23.1, 31.6)	118 (55.9)	83.1 (72.0, 95.9)
Child characteristics at age 7 years (*n* = 221)
Household income
At or below poverty	154 (69.4)	24.1 (21.0, 27.7)	148 (70.1)	88.0 (77.4, 100.1)
Above poverty	68 (30.6)	28.3 (21.9, 36.6)	63 (29.9)	72.4 (60.8, 86.4)
Soda consumption
< 1/week	119 (53.9)	24.7 (20.7, 29.4)	112 (53.1)	87.0 (75.1, 100.9)
1–6/week	83 (37.5)	27.0 (22.0, 33.1)	81 (38.4)	82.2 (70.3, 96.1)
≥ 1/day	19 (8.6)	21.6 (15.2, 30.7)	18 (8.5)	65.0 (41.5, 101.9)
Fast food consumption
< 1/week	108 (48.9)	24.8 (20.8, 29.5)	103 (48.8)	85.5 (74.0, 98.7)
1/week	93 (42.1)	23.9 (19.8, 28.8)	89 (42.2)	80.6 (67.6, 96.2)
>1/week	20 (9.0)	35.8 (21.3, 60.3)	19 (9.0)	81.5 (61.0, 109.0)
Average daily TV time
< 1 hr/day	43 (19.4)	29.3 (21.3, 40.3)	41 (19.4)	101.2 (78.1, 131.1)
1–2 hr/day	68 (30.8)	21.6 (17.2, 27.2)	64 (30.3)	78.5 (64.6, 95.3)
≥ 2 hr/day	110 (49.8)	26.2 (22.2, 30.9)	106 (50.3)	79.6 (69.0, 91.8)
Outdoor play time
< 1 hr/day	25 (11.5)	25.7 (18.7, 35.4)	24 (11.6)	80.5 (60.4, 107.4)
1–2 hr/day	126 (58.1)	26.2 (21.8, 31.4)	119 (57.5)	76.8 (66.6, 88.4)
≥ 2 hr/day	66 (30.4)	23.8 (19.5, 29.0)	64 (3.9)	96.1 (79.1, 116.7)
GM, geometric mean. All measures are lipid-adjusted (ng/g lipids).^***a***^Sum of 4 PBDE congeners: BDEs 47, 99, 100, and 153. ^***b***^Maternal BMI (kg/m^2^): underweight or normal < 24.9, overweight 25–29.9, obese ≥ 30.				

Written informed consent was obtained from mothers, and children provided verbal assent at 7 years of age. Study activities were approved by the Institutional Review Board at the University of California, Berkeley, and the Centers for Disease Control and Prevention (CDC).

*Procedure*. Bilingual, bicultural study staff conducted structured interviews in English or Spanish twice during pregnancy (mean ± SD = 13.4 ± 5.2 and 25.7 ± 2.1 weeks of gestation), soon after delivery, and when children were 2, 3.5, 5, and 7 years of age. Child weight (kilograms) and height (centimeters) were measured at each follow-up visit. Weight was measured once using a digital scale (Tanita 1582; Tanita Corporation, Arlington Heights, IL). Barefoot standing height was measured in triplicate using a stadiometer (Seca 222; Seca, Chino, CA) and the measures were averaged. Starting at 5 years, waist circumference was measured in triplicate by placing a measuring tape around the abdomen at the level of the iliac crest, parallel to the floor and measures were averaged. Maternal prepregnancy body mass index (BMI) was calculated using the mothers’ self-reported prepregnancy weight and height measured by stadiometer.

*PBDE exposure assessment*. Blood samples were collected by venipuncture from mothers during pregnancy (26.7 ± 2.6 weeks gestation, *n* = 219) or at delivery (*n* = 60), and from children at the 7-year visit (7.1 ± 0.3 years, *n* = 272). Samples were immediately processed and stored at –80°C until shipment on dry ice to the CDC in Atlanta, Georgia, where they were analyzed for 10 PBDE congeners (BDEs 17, 28, 47, 66, 85, 99, 100, 153, 154, and 183) by gas chromatography–isotope dilution–high resolution mass spectrometry (Sjödin et al. 2004). The individual pentaBDE mixture congeners (BDEs 47, 99, 100, and 153) with detection frequencies ≥ 90% and the sum of these congeners were selected as the primary exposure. PBDE concentrations were adjusted for serum lipid levels and expressed on a serum lipid basis (nanograms per gram lipid). Total serum lipid concentrations were estimated based on triglycerides and total cholesterol measured using standard enzymatic methods (Roche Chemicals, Indianapolis, IN) (Phillips et al. 1989). The limits of detection (LODs) for BDE-47 ranged from 0.3 to 2.6 ng/g lipid for maternal samples, and 0.4 to 0.8 ng/g lipid for child samples. For all other congeners, LODs ranged between 0.2 and 0.7 ng/g lipid for maternal samples and 0.3 and 5.6 ng/g lipid for child samples. Laboratory blanks and spikes were included in each run.

Values below the LODs were assigned the machine-read value if a signal was detected and otherwise imputed at random based on a log-normal probability distribution whose parameters were determined using maximum likelihood estimation (Lubin et al. 2004).

*Other laboratory analyses*. The CDC also measured *p,p´*-dichl​orodipheny​ltrich​loroet​hane (DDT) and *p,p´*-dichlorodiphenyldichloroethylene (DDE) in maternal serum collected around the 26th week of gestation using gas chromatography–high resolution mass spectrometry (Eskenazi et al. 2006). TSH was measured by immunochemiluminometric assay (Quest Diagnostics’ Nichols Institute; San Juan Capistrano, CA) and free T_4_ using direct equilibrium dialysis followed by radioimmunoassay (Bayer ADVIA Centaur system; Siemens Healthcare Diagnostics, Deerfield, IL) (Nelson and Tomei 1988). Per the California Department of Health Services Neonatal Genetics Disease Screening Program, dried blood spots collected from newborns were analyzed for TSH using solid-phase, time-resolved sandwich fluoroimmunoassay (AutoDELPHIA system; PerkinElmer, Wellesley, MA).

*Data analysis*. We examined the distributions of PBDE concentrations and found them to be strongly right skewed; thus, they were log_10_ transformed to reduce the influence of outliers. We categorized PBDE concentration variables by quintiles to investigate nonmonotonic exposure–response relationships. We used Pearson correlations to assess the correlation between concentrations of the four PBDE congeners and between prenatal and child (at age 7 years) PBDE blood concentrations. Age- and sex-specific BMI *z*-scores and percentiles were computed using 2000 CDC growth charts (Kuczmarski et al. 2002). BMI < 85th percentile, between the 85th and 95th percentiles, and ≥ 95th percentile were defined as normal BMI, overweight, and obese, respectively.

We used multivariable regression to examine associations between maternal concentrations for each individual pentaBDE congener (BDEs 47, 99, 100, and 153) and for the sum of these four congeners (Σ4PBDE) and anthropometric measurements (BMI and waist circumference) of the children at each age (2, 3.5, 5 years). We also constructed separate models to examine associations between anthropometric measurements at 7 years and maternal as well as child PBDE concentrations at 7 years. Linear regression was used to examine the relationship of PBDE concentrations and continuous outcomes (BMI and waist circumference *z*-score), and logistic regression was used to examine categorical outcomes (BMI categories). Generalized estimating equation (GEE) models were used to examine the population average association between prenatal PBDE exposure and BMI *z*-score at ages 2, 3.5, 5, or 7 years (*n* = 224, average number of observations = 3.8), and (in separate models) waist circumference *z*-scores at ages 5 and 7 years (*n* = 224); standard errors and 95% confidence intervals (CIs) were estimated by using a robust (Huber–White) variance estimate.

Potential confounding variables were selected *a priori* based on the childhood obesity literature (Berkey et al. 2000; Ebbeling et al. 2002; Hernández et al. 1999; Levin and Govek 1998) and a directed acyclic graph (DAG) (see Supplemental Material, Figure S1). We considered the following as potential confounders: maternal age, education, prepregnancy BMI, years resided in the United States at enrollment, gestational weight gain, parity; family poverty status; and child sex, gestational age at delivery, duration of breastfeeding, and childhood dietary and physical activity characteristics (e.g., intake of soda, sweetened beverages, fast food, sweet and salty snacks, and time spent watching television and playing outside at 7 years). Based on the DAG, final models included the following covariates: maternal age, education, prepregnancy BMI, years resided in the United States, gestational weight gain, and poverty during pregnancy; and child gestational age at delivery, duration of breast feeding, and fast food and soda consumption at age 7 years.

We conducted a number of sensitivity analyses. Though lipid-adjusted log_10_ PBDE concentrations were the primary independent variable, we also considered wet-weight PBDE concentrations (wet-weight picograms per gram serum) adjusting for serum lipids (milligrams per deciliter) (Chevrier 2013). We also considered unlogged PBDE concentrations (nanograms per gram lipid). We also reran models of body mass of the children at 7 years controlling for both maternal and child PBDE concentrations in the same model. We also used categorical PBDE concentration variables (quintiles) to investigate nonmonotonic exposure–response relationships. Because maternal pregnancy PBDE serum concentrations have previously shown associations with TSH in CHAMACOS participants (Chevrier et al. 2010) and thyroid function is a determinant of obesity, we conducted sensitivity analyses to determine whether thyroid hormone could be a potential mediator (Cole and Hernán 2002). Specifically, we modeled associations of pregnancy PBDE with body mass at age 7 years adjusting separately for maternal TSH, maternal free T_4_, and neonatal TSH while controlling for confounders of the thyroid hormone–obesity relations (Cole and Hernán 2002). Although in our DAG we considered birth weight as potentially on the causal pathway (Harley et al. 2011) and that it might result in spurious findings if controlled for (Hernández-Díaz et al. 2006), we conducted sensitivity analysis controlling for birth weight. Because our study population has relatively high exposure to DDT and DDE (Bradman et al. 2005), we reran our main models controlling for DDT/E.

We adjusted for potential selection bias in the reduced sample by applying weights equal to inverse probability of being included in the final analysis. Weights were determined using a SuperLearner algorithm that minimizes cross-validated risks based on a loss function (van der Laan et al. 2007). Main effects and interactions were considered statistically significant at *p* < 0.05, and *p* < 0.10 based on two-tailed tests, respectively. All analyses were conducted with STATA version 12.1 (StataCorp, College Station, TX).

## Results

*Participants’ characteristics*. Table 1 presents demographic characteristics and maternal and child Σ4PBDE serum concentrations. At enrollment, mean (± SD) age of mothers was 25.7 ± 5.0 years. Before pregnancy, 64.3% of mothers were overweight or obese, and 54.5% gained more weight than recommended during pregnancy (American Congress of Obstetricians and Gynecologists 2013). The percent of the children who were obese increased from age 2 to 7 years [16.4% at age 2 (*n* = 207), 29.1% at age 3.5 (*n* = 203), 33.0% at age 5 (*n* = 209), 34.4% at age 7 (*n* = 221) were obese] (see Supplemental Material, Table S1). At age 7, 18.6% of children were overweight.

*PBDE concentrations*. The geometric means and 95% CIs for maternal and child Σ4PBDE concentrations were 25.35 (22.42, 28.65) and 83.03 (74.80, 92.17) ng/g lipid, respectively, with the largest contribution from BDE-47 (see Supplemental Material, Table S2). BDEs 47, 99, and 100 were highly correlated with each other and with Σ4PBDE (*r* > 0.90) in mothers and in children. BDE-153 was less strongly correlated with other congeners (0.72–0.89 in mothers and 0.65–0.76 in children) (see Supplemental Material, Table S3). As we previously reported, the correlation between maternal and child Σ4PBDE was 0.27 (*p* < 0.01) (Bradman et al. 2012).

**Figure 1:**
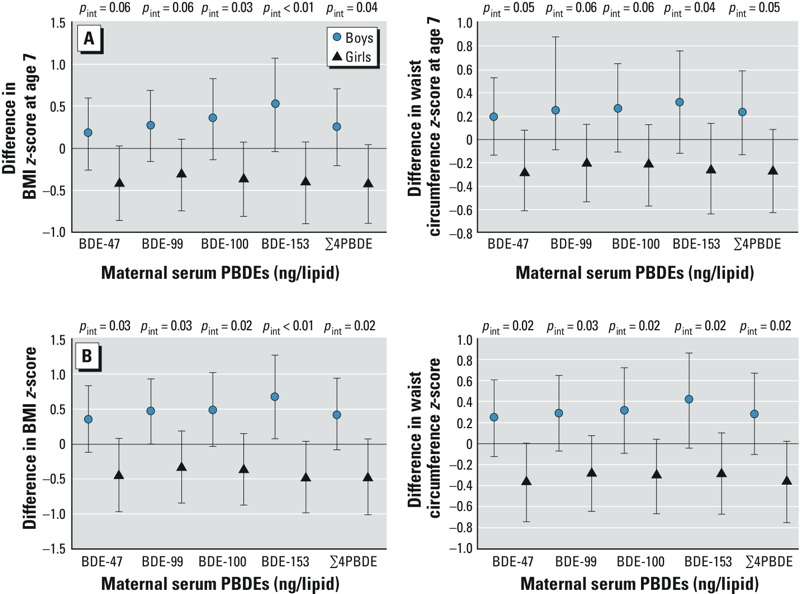
Point estimates and 95% CIs from (*A*) regression of maternal PBDE concentrations and anthropometric measurements at age 7 years, and (*B*) GEE model estimates of overall associations between 10-fold increases in maternal PBDE concentrations and repeated anthropometric measures (ages 2, 3.5, 5, and 7 years), with effect modification by sex, controlling for maternal age, education, prepregnancy BMI, years lived in the United States, gestational weight gain, poverty during pregnancy; and child gestational age, duration of breastfeeding, and fast food and soda consumption at age 7. *p*_int_, *p*-value for interaction.

*Maternal PBDE concentrations and measures of child body mass*. Maternal serum concentrations for each pentaBDE congener (BDEs 47, 99, 100, and 153) and for Σ4PBDE were not associated with any measures of the child’s body mass at age 7 years. Specifically, maternal serum Σ4PBDE concentration was not associated with the BMI *z*-score (β_adjusted_ = –0.08; 95% CI: –0.41, 0.25), waist circumference *z*-score (β_adjusted_ = –0.02; 95% CI: –2.45, 0.28), or the odds of being overweight at age 7 (OR_adjusted_ = 0.82; 95% CI: 0.38, 1.79) (see Supplemental Material, Table S4). (Point estimates were reported in log_10_ scale.) However, we observed evidence of effect modification by sex for all four congeners and their sum (Figure 1A). Each 10-fold increase in maternal serum Σ4PBDE concentration was associated with a significant 0.41-unit decrease in BMI *z*-score in girls (95% CI: –0.87, –0.05) but with a nonsignificant increase in BMI *z*-score in boys (β_adjusted_ = 0.26; 95% CI: –0.19, 0.72; *p*_interaction_ = 0.04). Waist circumference *z*-scores and obesity status models showed similar, significant effect modification by sex (see Supplemental Material, Table S5), though overweight status models did not (data not shown). Likewise, GEE models of associations with repeated measures of the outcomes from 2 to 7 years showed no overall association of maternal serum Σ4PBDE or individual penta-congener concentrations with BMI or waist circumference *z*-score (β_adjusted_ = –0.02; 95% CI: –0.44, 0.39; β_adjusted_ = –0.002; 95% CI: –0.29, 0.29 for Σ4PBDE, data not shown), but there was effect modification by sex for both outcomes (Figure 1B). Results for the above models did not change when controlling for child age 7 PBDE concentrations (data not shown). See Supplemental Material, Figure S2, for crude associations between maternal and child Σ4PBDE concentrations and BMI *z*-score, with regression lines for boys and girls at each age. Starting from age 2 years, an association between maternal BDE-153 exposure and BMI was observed with effect modification by child sex (see Supplemental Material, Table S6). At age 3.5 years, the relationship between maternal BDE-153 levels and BMI *z*-score was significantly negative in girls (β_adjusted_ = –0.64; 95% CI: –1.23, –0.06) and significantly positive in boys (β_adjusted_ = 0.99; 95% CI: 0.32, 1.66) (*p*_interaction_ < 0.01) (see Supplemental Material, Table S6).

**Figure 2:**
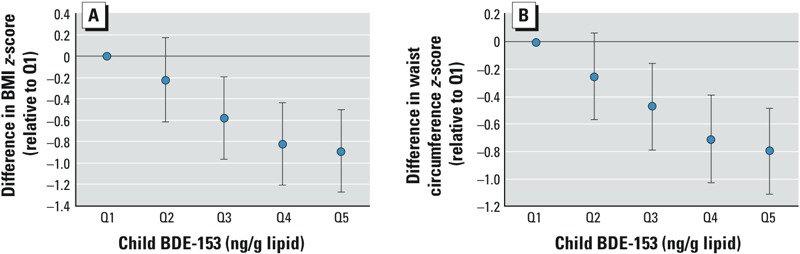
The point estimates and 95% CIs for each quintile (Q) of child BDE-153 (ranges were ≥ 6.19, 6.19–9.01, 9.01–13.25, 13.25–22.36, ≥ 22.36 ng/g lipid) for (*A*) BMI *z*-score and (*B*) waist circumference *z*-score controlling for maternal age, education, prepregnancy BMI, years lived in the United States, gestational weight gain, poverty during pregnancy; and child gestational age, duration of breast feeding, and fast food and soda consumption at age 7 years.

*Child PBDE concentrations and measures of body mass*. Table 2 shows the cross-sectional association of children’s age 7 serum Σ4PBDE concentrations and body mass measures. Child Σ4PBDE concentrations were associated with significantly lower in BMI (β_adjusted_ = –0.44; 95% CI: –0.83, –0.06) and waist circumference *z*-scores (β_adjusted_ = –0.35; 95% CI: –0.66, –0.04), and a significant decrease in odds of being overweight [adjusted odds ratio (OR_adjusted_) = 0.36 for each 10-fold increase in Σ4PBDE; 95% CI: 0.14, 0.94]. In particular, BDE-153 concentration was associated with significantly lower in BMI (β_adjusted_ = –1.15; 95% CI: –1.53, –0.77) and waist circumference *z*-scores (β_adjusted_ = –0.95; 95% CI: –1.26, –0.64). Figure 2 presents the relationship of BMI and waist circumference *z*-scores at age 7 with concurrent child BDE-153 concentrations (quintiles). We observed a monotonic decrease with increasing quartiles of exposure. We did not find evidence of effect modification by sex with Σ4PBDE or the individual penta-congeners in child sera (data not shown).

**Table 2:** Unadjusted and adjusted associations between 10-fold increase in child serum concentrations of penta-PBDE (log_10_) and child body mass outcomes at age 7 years.

	BMI *z*-scoreβ (95% CI)	Waist circumference β (95% CI)	Overweight^*a*^OR (95% CI)	Obese^*b*^OR (95% CI)
Crude
BDE-47	–0.23 (–0.61, 0.15)	–0.17 (–0.48, 0.15)	0.63 (0.29, 1.35)	0.84 (0.39, 1.89)
BDE-99	–0.25 (–0.60, 0.10)	–0.20 (–0.49, 0.09)	0.66 (0.33, 1.34)	0.75 (0.36, 1.57)
BDE-100	–0.29 (–0.68, 0.10)	–0.23 (–0.56, 0.09)	0.55 (0.25, 1.21)	0.79 (0.35, 1.78)
BDE-153	–1.23 (–1.63, –0.84)*	–1.02 (–1.35, –0.69)*	0.11 (0.04, 0.31)*	0.15 (0.05, 0.37)*
Σ4PBDE^*c*^	–0.45 (–0.85, –0.04)*	–0.35 (–0.68, 0.01)*	0.46 (0.19, 1.05)**	0.62 (0.26, 1.46)
Adjusted^*d*^
BDE-47	–0.25 (–0.61, 0.11)	–0.19 (–0.49, 0.09)	0.50 (0.21, 1.21)	0.78 (0.31, 1.99)
BDE-99	–0.26 (–0.59, 0.07)	–0.21 (–0.49, 0.06)	0.53 (0.23, 1.21)	0.69 (0.29, 1.65)
BDE-100	–0.31 (–0.68, 0.06)**	–0.27 (–0.57, –0.04)**	0.40 (0.16, 1.03)**	0.69 (0.26, 1.82)
BDE-153	–1.15 (–1.53, –0.77)*	–0.95 (–1.26, –0.64)*	0.08 (1.03, 0.27)*	1.59 (0.61, 4.16)
Σ4PBDE^*c*^	0.44 (–0.83, –0.06)*	–0.35 (–0.66, –0.04)*	0.36 (0.14, 0.94)*	1.09 (0.49, 2.46)
OR, odds ratio. ^***a***^Overweight: age- and sex-specific BMI ≥ the 85th percentile but < the 95th percentile. ^***b***^Obese: age- and sex-specific BMI ≥ the 95th percentile. ^***c***^Sum of 4 penta-BDE congeners: BDEs 47, 99, 100, and 153. ^***d***^Controlling for maternal age, maternal education, maternal years in USA, maternal prepregnancy BMI, maternal gestational weight gain, poverty during pregnancy, gestational age, breastfeeding duration, and soda and fast food consumption at 7 years of age. *p < 0.05 statistically significant. **p < 0.1.				

*Sensitivity analyses*. Results for final models were qualitatively similar when we expressed PBDE concentrations on a serum volume basis controlling for serum lipid levels; when using unlogged PBDE concentrations (nanograms per gram lipid); when maternal and child PBDE concentrations were entered into the same model; and when we controlled for maternal TSH and free T_4_, neonatal TSH, child birth weight, and maternal DDT/E (data not shown). When we adjusted for potential selection bias in the reduced sample by applying weights equal to inverse probability of being included in the final analysis, our results did not change (data not shown).

## Discussion

In this study of 7-year-old Latino children in an agricultural California community, although we did not observe significant overall associations between maternal prenatal pentaBDE concentrations and measures of child’s body mass, we did demonstrate significant effect modifications by sex, with generally negative associations for girls but positive associations for boys. We also demonstrated that children’s serum PBDE concentrations at age 7 years, specifically BDE-153 concentration, were negatively associated with concurrent BMI *z*-score, waist circumference *z*-score, and being overweight for both sexes combined. Although we previously reported an association between maternal PBDE levels and child birth weight (Harley et al. 2011), controlling for birth weight did not alter our above findings, suggesting an association of PBDEs on child weight independent of birth weight.

To our knowledge, no previous studies in humans have examined prenatal PBDE exposure and child BMI. However, a single study in rats showed a modest increase (approximately 7%) in body weight in both sexes with perinatal pentaBDE exposure where the animals were treated by gavage with PBDEs that are commonly found in humans (i.e., BDEs 47, 99, 100, and 153) (Bondy et al. 2013). Previous human and animal studies of postnatal exposure have produced inconsistent results. Rodent studies of postnatal oral pentaBDE exposure have not shown associations with body weight (Ernest et al. 2012; Fowles et al. 1994; Stoker et al. 2004). In cross-sectional studies of human adults, one study reported a positive association between BMI and serum concentrations of BDEs 47, 99, and 100, but no relationship with BDE-153 (Turyk et al. 2010); and another study reported inverse associations with serum BDE-153 levels (as we observed here) but no association with other congeners (Lim et al. 2008). In a study of PBDEs and body mass in girls 6 to 9 years of age, Windham et al. (2010) reported that total child serum PBDE (sum of BDEs 28, 47, 99, 100, and 153), and BDEs 154 and 153 levels were significantly lower in overweight and obese girls relative to those with normal BMI).

The relationship between serum PBDE concentration and obesity is likely to be complex. We hypothesized that reverse causality, particularly for the higher lipophilic congeners, may explain, at least in part, the inverse relationship between child BDEs blood concentrations and body mass. Serum PBDE concentration is determined by the amount and route of exposure, homeostasis between tissues and extracellular fluids, and rates of metabolism and excretion (Staskal et al. 2006). Because each PBDE congener has different absorption rates from the gastrointestinal tract, lipophilicity, adipose tissue/serum concentration ratios, and elimination rates, individual serum congener measurements may not always reflect the level of external exposure or total body congener amount, and therefore may not be comparable (Bondy et al. 2011, 2013; Sanders et al. 2006; Staskal et al. 2006). For example, in our study population, the prevalence of obesity increased with age. As suggested previously for PBDEs and for other lipophilic compounds (Chevrier 2013), this weight gain might create an additional adipose tissue reservoir for storage of PBDEs, leading to diluted, and thus lower, concentrations in heavier individuals (Glynn et al. 2003). Thus, reverse causality may explain the inverse association of children’s serum BDE-153 concentration with children’s BMI *z*-score, waist circumference *z*-score, and overweight/obesity status, especially because BDE-153 is the most lipophilic, has the highest bioaccumulation capacity and adipose tissue/serum concentration ratio, and the lowest rate of metabolism and excretion (Bondy et al. 2011; Sanders et al. 2006; Staskal et al. 2006) compared with the other PBDEs measured. A cross-sectional study in adults that reported a negative association only between serum BDE-153 levels and BMI provides additional support (Lim et al. 2008).

In contrast, the observed relationships with *in utero* exposure cannot be readily explained by reverse causality. It has been proposed that *in utero* exposure to environmental chemicals can result in alteration of developmental programming of central endocrine regulatory systems, which in turn could result in higher or lower susceptibility to obesity later in life (Baillie-Hamilton 2002; Grün et al. 2006; Hanson and Gluckman 2008). Although we found little evidence to support a general “obesogenic” effect of *in utero* PBDE exposure, we found sex-specific differences—namely, positive associations between *in utero* PBDE exposure and childhood body mass in boys but an inverse relationship in girls. We previously reported in CHAMACOS that maternal urinary BPA concentrations were also negatively associated with BMI *z*-score of their daughters at 9 years of age (Harley et al. 2013). These findings remained unchanged when we controlled for maternal PBDE concentrations. PBDEs act on steroid receptors and may disrupt estrogen and androgen signaling in both sexes (Meerts et al. 2001; Stoker et al. 2005), but the sexually dimorphic nature of sex steroid milieu and distribution of their receptors in the central nervous system and adipose tissue may have contributed to the apparent sex-specific association with *in utero* PBDE exposure (Grün and Blumberg 2007). Although the exact pathophysiology has not been well described, sex-specific *in utero* effects of endocrine-disrupting compounds with weight have been reported by other studies (Karmaus et al. 2009; Petreas et al. 2011; Vilahur et al. 2013; Wolff et al. 2008).

This study has many strengths. The CHAMACOS study is a longitudinal birth cohort with PBDE concentrations measured in both maternal pregnancy and child serum and with multiple measures of child anthropometrics. Information on many potential confounders was available, and the population is relatively homogeneous with regard to race, socioeconomic status, and diet, which can reduce uncontrolled confounding. However, the results of this study may not be generalizable to the entire U.S. population due to the specific demographic makeup of the study population, the particularly high rates of childhood overweight and obesity in CHAMACOS (Ogden et al. 2012), and the relatively high child PBDE concentrations in California (Windham et al. 2010). One of the limitations of our study is the lack of detailed dietary information at each age of follow-up. Lack of measurement of hydroxyl metabolites of PBDEs, which are biologically active and may also disturb sex steroid receptor signaling, is another limitation. Although use of self-reported maternal prepregnancy weight is another weakness, this method was previously validated (Shin et al. 2014).

## Conclusion

We evaluated potential obesogenic effects of *in utero* and postnatal exposure to PBDE in the CHAMACOS longitudinal birth cohort through 7 years of age. Our findings suggest that associations between *in utero* PBDE exposure and body weight at age 7 differ between boys and girls. Our analysis also shows an inverse association of child serum BDE-153, but not other pentaBDE congeners, with concurrent markers of body mass at age 7 years, though this may be attributable to reverse causality. Future studies should confirm this observed effect modification by sex and further examine whether the effect modification by sex observed in this study is modified by the changes in the hormonal environment through puberty. Finally, because PBDE flame retardants have been replaced by other potentially endocrine-disrupting chemicals, the obesogenic characteristics of these chemicals should also be investigated.

## Supplemental Material

(1.8 MB) PDF

## References

[r1] American Congress of Obstetricians and Gynecologists. (2013). ACOG Committee opinion no 548: weight gain during pregnancy.. Obstet Gynecol.

[r2] Baillie-Hamilton PF (2002). Chemical toxins: a hypothesis to explain the global obesity epidemic.. J Altern Complement Med.

[r3] Berkey CS, Rockett HR, Field AE, Gillman MW, Frazier AL, Camargo CA (2000). Activity, dietary intake, and weight changes in a longitudinal study of preadolescent and adolescent boys and girls.. Pediatrics.

[r4] Besis A, Samara C (2012). Polybrominated diphenyl ethers (PBDEs) in the indoor and outdoor environments—a review on occurrence and human exposure.. Environ Pollut.

[r5] Bondy GS, Gaertner D, Cherry W, MacLellan E, Coady L, Arnold DL (2011). Brominated diphenyl ether (BDE) levels in liver, adipose, and milk from adult and juvenile rats exposed by gavage to the DE-71 technical mixture.. Environ Toxicol.

[r6] Bondy GS, Lefebvre DE, Aziz S, Cherry W, Coady L, MacLellan E (2013). Toxicologic and immunologic effects of perinatal exposure to the brominated diphenyl ether (BDE) mixture DE-71 in the Sprague-Dawley rat.. Environ Toxicol.

[r7] Booth ML, Wake M, Armstrong T, Chey T, Hesketh K, Mathur S (2001). The epidemiology of overweight and obesity among Australian children and adolescents, 1995–97.. Aust NZ J Public Health.

[r8] Bradman A, Castorina R, Sjödin A, Fenster L, Jones RS, Harley KG (2012). Factors associated with serum polybrominated diphenyl ether (PBDE) levels among school-age children in the CHAMACOS cohort.. Environ Sci Technol.

[r9] Bradman A Eskenazi B Barr DB Bravo R Castorina R Chevrier J 2005 Organophosphate urinary metabolite levels during pregnancy and after delivery in women living in an agricultural community. Environ Health Perspect 113 1802 1807; 10.1289/ehp.7894 16330368 PMC1314925

[r10] Chevrier J (2013). Invited commentary: Maternal plasma polybrominated diphenyl ethers and thyroid hormones—challenges and opportunities.. Am J Epidemiol.

[r11] Chevrier J Harley KG Bradman A Gharbi M Sjödin A Eskenazi B 2010 Polybrominated diphenyl ether (PBDE) flame retardants and thyroid hormone during pregnancy. Environ Health Perspect 118 1444 1449; 10.1289/ehp.1001905 20562054 PMC2957927

[r12] Cole SR, Hernán MA (2002). Fallibility in estimating direct effects.. Int J Epidemiol.

[r13] Ebbeling CB, Pawlak DB, Ludwig DS (2002). Childhood obesity: public-health crisis, common sense cure.. Lancet.

[r14] Ernest SR, Wade MG, Lalancette C, Ma YQ, Berger RG, Robaire B (2012). Effects of chronic exposure to an environmentally relevant mixture of brominated flame retardants on the reproductive and thyroid system in adult male rats.. Toxicol Sci.

[r15] Eskenazi B, Bradman A, Gladstone EA, Jaramillo S, Birch K, Holland NT (2003). CHAMACOS, a longitudinal birth cohort study: lessons from the fields.. J Children’s Health.

[r16] Eskenazi B Fenster L Castorina R Marks AR Sjödin A Rosas LG 2011 A comparison of PBDE serum concentrations in Mexican and Mexican-American children living in California. Environ Health Perspect 119 1442 1448; 10.1289/ehp.1002874 21498147 PMC3230428

[r17] Eskenazi B Harley K Bradman A Weltzien E Jewell NP Barr DB 2004 Association of *in utero* organophosphate pesticide exposure and fetal growth and length of gestation in an agricultural population. Environ Health Perspect 112 1116 1124; 10.1289/ehp.6789 15238287 PMC1247387

[r18] Eskenazi B, Marks AR, Bradman A, Fenster L, Johnson C, Barr DB (2006). In utero exposure to dichlorodiphenyltrichloroethane (DDT) and dichlorodiphenyldichloroethylene (DDE) and neurodevelopment among young Mexican American children.. Pediatrics.

[r19] Fowles JR, Fairbrother A, Baecher-Steppan L, Kerkvliet NI (1994). Immunologic and endocrine effects of the flame-retardant pentabromodiphenyl ether (DE-71) in C57BL/6J mice.. Toxicology.

[r20] Geyer HJ, Schramm KW, Darnerud PO, Aune M, Feicht A, Fried KW (2004). Terminal elimination half-lives of the brominated flame retardants TBBPA, HBCD, and lower brominates PBDEs in humans.. Organohalogen Compounds.

[r21] Glynn AW Granath F Aune M Atuma S Darnerud PO Bjerselius R 2003 Organochlorines in Swedish women: determinants of serum concentrations. Environ Health Perspect 111 349 355; 10.1289/ehp.5456 12611665 PMC1241393

[r22] Grün F, Blumberg B (2007). Perturbed nuclear receptor signaling by environmental obesogens as emerging factors in the obesity crisis.. Rev Endocr Metab Disord.

[r23] Grün F, Watanabe H, Zamanian Z, Maeda L, Arima K, Cubacha R (2006). Endocrine-disrupting organotin compounds are potent inducers of adipogenesis in vertebrates.. Mol Endocrinol.

[r24] Hagmar L, Björk J, Sjödin A, Bergman A, Erfurth EM (2001). Plasma levels of persistent organohalogens and hormone levels in adult male humans.. Arch Environ Health.

[r25] Hale RC, Alaee M, Manchester-Neesvig JB, Stapleton HM, Ikonomou MG (2003). Polybrominated diphenyl ether flame retardants in the North American environment.. Environ Int.

[r26] Hallgren S, Sinjari T, Håkansson H, Darnerud PO (2001). Effects of polybrominated diphenyl ethers (PBDEs) and polychlorinated biphenyls (PCBs) on thyroid hormone and vitamin A levels in rats and mice.. Arch Toxicol.

[r27] Hanson MA, Gluckman PD (2008). Developmental origins of health and disease: new insights.. Basic Clin Pharmacol Toxicol.

[r28] Harley KG, Chevrier J, Aguilar Schall R, Sjödin A, Bradman A, Eskenazi B (2011). Association of prenatal exposure to polybrominated diphenyl ethers and infant birth weight.. Am J Epidemiol.

[r29] Harley KG, Gunier RB, Kogut K, Johnson C, Bradman A, Calafat AM (2013). Prenatal and early childhood bisphenol A concentrations and behavior in school-aged children.. Environ Res.

[r30] Herbstman JB Sjödin A Kurzon M Lederman SA Jones RS Rauh V 2010 Prenatal exposure to PBDEs and neurodevelopment. Environ Health Perspect 118 712 719; 10.1289/ehp.0901340 20056561 PMC2866690

[r31] Hernández B, Gortmaker SL, Colditz GA, Peterson KE, Laird NM, Parra-Cabrera S (1999). Association of obesity with physical activity, television programs and other forms of video viewing among children in Mexico City.. Int J Obes Relat Metab Disord.

[r32] Hernández-Díaz S, Schisterman EF, Hernán MA (2006). The birth weight “paradox” uncovered?. Am J Epidemiol.

[r33] Hoppe AA, Carey GB (2007). Polybrominated diphenyl ethers as endocrine disruptors of adipocyte metabolism.. Obesity (Silver Spring).

[r34] Janesick A, Blumberg B (2011a). Endocrine disrupting chemicals and the developmental programming of adipogenesis and obesity.. Birth Defects Res C Embryo Today.

[r35] Janesick A, Blumberg B (2011b). Minireview: PPARγ as the target of obesogens.. J Steroid Biochem Mol Biol.

[r36] Karmaus W, Osuch JR, Eneli I, Mudd LM, Zhang J, Mikucki D (2009). Maternal levels of dichlorodiphenyl-dichloroethylene (DDE) may increase weight and body mass index in adult female offspring.. Occup Environ Med.

[r37] Kuczmarski RJ Ogden CL Guo SS Grummer-Strawn LM Flegal KM Mei Z 2002 2000 CDC growth charts for the United States: methods and development. Vital Health Stat 11(246 1 190 12043359

[r38] Kuriyama SN, Wanner A, Fidalgo-Neto AA, Talsness CE, Koerner W, Chahoud I (2007). Developmental exposure to low-dose PBDE-99: Tissue distribution and thyroid hormone levels.. Toxicology.

[r39] Legler J, Brouwer A (2003). Are brominated flame retardants endocrine disruptors?. Environ Int.

[r40] Legler J, Hamers T, van Eck van der Sluijs-van de Bor M, Schoeters G, van der Ven L, Eggesbo M (2011). The OBELIX project: early life exposure to endocrine disruptors and obesity.. Am J Clin Nutr.

[r41] Levin BE, Govek E (1998). Gestational obesity accentuates obesity in obesity-prone progeny.. Am J Physiol.

[r42] Li L, Xie S, Cai H, Bai X, Xue Z (2008). Quantitative structure–property relationships for octanol–water partition coefficients of polybrominated diphenyl ethers.. Chemosphere.

[r43] Lim JS, Lee DH, Jacobs DR (2008). Association of brominated flame retardants with diabetes and metabolic syndrome in the U.S. population, 2003–2004.. Diabetes Care.

[r44] Lobstein T (2004). The prevention of obesity in children.. Pediatr Endocrinol Rev.

[r45] Lorber M (2008). Exposure of Americans to polybrominated diphenyl ethers.. J Expo Sci Environ Epidemiol.

[r46] Lubin JH Colt JS Camann D Davis S Cerhan JR Severson RK 2004 Epidemiologic evaluation of measurement data in the presence of detection limits. Environ Health Perspect 112 1691 1696; 10.1289/ehp.7199 15579415 PMC1253661

[r47] Meerts IA, Letcher RJ, Hoving S, Marsh G, Bergman A, Lemmen JG (2001). *In vitro* estrogenicity of polybrominated diphenyl ethers, hydroxylated PBDEs, and polybrominated bisphenol A compounds.. Environ Health Perspect.

[r48] Nelson JC, Tomei RT (1988). Direct determination of free thyroxin in undiluted serum by equilibrium dialysis/radioimmunoassay.. Clin Chem.

[r49] Noyes PD, Hinton DE, Stapleton HM (2011). Accumulation and debromination of decabromodiphenyl ether (BDE-209) in juvenile fathead minnows (*Pimephales promelas*) induces thyroid disruption and liver alterations.. Toxicol Sci.

[r50] Ogden CL, Carroll MD, Kit BK, Flegal KM (2012). Prevalence of obesity in the United States, 2009–2010.. NCHS Data Brief.

[r51] Petreas M, Nelson D, Brown FR, Goldberg D, Hurley S, Reynolds P (2011). High concentrations of polybrominated diphenylethers (PBDEs) in breast adipose tissue of California women.. Environ Int.

[r52] Phillips DL, Pirkle JL, Burse VW, Bernert JT, Henderson LO, Needham LL (1989). Chlorinated hydrocarbon levels in human serum: effects of fasting and feeding.. Arch Environ Contam Toxicol.

[r53] Popkin BM, Doak CM (1998). The obesity epidemic is a worldwide phenomenon.. Nutr Rev.

[r54] Sanders JM, Lebetkin EH, Chen LJ, Burka LT (2006). Disposition of 2,2´,4,4´,5,5´-hexabromodiphenyl ether (BDE153) and its interaction with other polybrominated diphenyl ethers (PBDEs) in rodents.. Xenobiotica.

[r55] Shin D, Chung H, Weatherspoon L, Song WO (2014). Validity of prepregnancy weight status estimated from self-reported height and weight.. Matern Child Health J.

[r56] Sjödin A, Jones RS, Lapeza CR, Focant JF, McGahee EE, Patterson DG (2004). Semiautomated high-throughput extraction and cleanup method for the measurement of polybrominated diphenyl ethers, polybrominated biphenyls, and polychlorinated biphenyls in human serum.. Anal Chem.

[r57] Sjödin A, Päpke O, McGahee E, Focant JF, Jones RS, Pless-Mulloli T (2008). Concentration of polybrominated diphenyl ethers (PBDEs) in household dust from various countries.. Chemosphere.

[r58] Staskal DF, Hakk H, Bauer D, Diliberto JJ, Birnbaum LS (2006). Toxicokinetics of polybrominated diphenyl ether congeners 47, 99, 100, and 153 in mice.. Toxicol Sci.

[r59] Stoker TE, Cooper RL, Lambright CS, Wilson VS, Furr J, Gray LE (2005). In vivo and in vitro anti-androgenic effects of DE-71, a commercial polybrominated diphenyl ether (PBDE) mixture.. Toxicol Appl Pharmacol.

[r60] Stoker TE, Laws SC, Crofton KM, Hedge JM, Ferrell JM, Cooper RL (2004). Assessment of DE-71, a commercial polybrominated diphenyl ether (PBDE) mixture, in the EDSP male and female pubertal protocols.. Toxicol Sci.

[r61] Turyk ME, Anderson HA, Steenport D, Buelow C, Imm P, Knobeloch L (2010). Longitudinal biomonitoring for polybrominated diphenyl ethers (PBDEs) in residents of the Great Lakes Basin.. Chemosphere.

[r62] Turyk ME Persky VW Imm P Knobeloch L Chatterton R Anderson HA 2008 Hormone disruption by PBDEs in adult male sport fish consumers. Environ Health Perspect 116 1635 1641; 10.1289/ehp.11707 19079713 PMC2599756

[r63] van der Laan MJ, Polley EC, Hubbard AE. 2007 Super learner. Stat Appl Genet Mol Biol 6:Article25; 10.2202/1544-6115.1309 17910531

[r64] Vilahur N, Molina-Molina JM, Bustamante M, Murcia M, Arrebola JP, Ballester F (2013). Male specific association between xenoestrogen levels in placenta and birthweight.. Environ Int.

[r65] Windham GC, Pinney SM, Sjodin A, Lum R, Jones RS, Needham LL (2010). Body burdens of brominated flame retardants and other persistent organo-halogenated compounds and their descriptors in US girls.. Environ Res.

[r66] Wolff MS Engel SM Berkowitz GS Ye X Silva MJ Zhu C 2008 Prenatal phenol and phthalate exposures and birth outcomes. Environ Health Perspect 116 1092 1097; 10.1289/ehp.11007 18709157 PMC2516577

[r67] Wong F, Cousins IT, MacLeod M (2013). Bounding uncertainties in intrinsic human elimination half-lives and intake of polybrominated diphenyl ethers in the North American population.. Environ Int.

[r68] Wu N McClean MD Brown P Aschengrau A Webster TF 2009 Participant experiences in a breastmilk biomonitoring study: a qualitative assessment. Environ Health 8 4; 10.1186/1476-069X-8-4 19226469 PMC2649062

[r69] Zhou T, Taylor MM, DeVito MJ, Crofton KM (2002). Developmental exposure to brominated diphenyl ethers results in thyroid hormone disruption.. Toxicol Sci.

[r70] Zota AR, Rudel RA, Morello-Frosch RA, Brody JG (2008). Elevated house dust and serum concentrations of PBDEs in California: unintended consequences of furniture flammability standards?. Environ Sci Technol.

