# Erratum: “*In Utero* and Childhood Polybrominated Diphenyl Ether Exposures and Body Mass at Age 7 Years: The CHAMACOS Study”

**DOI:** 10.1289/ehp.124-A68

**Published:** 2016-04-01

**Authors:** Ayca Erkin-Cakmak, Kim G. Harley, Jonathan Chevrier, Asa Bradman, Katherine Kogut, Karen Huen, Brenda Eskenazi

Environ Health Perspect 123(6):636–642 (2015), http://dx.doi.org/10.1289/ehp.1408417

In Figure 1A, which represents the association between the difference in BMI *z*-score at age 7 and maternal serum PBDEs (ng/lipid), the line of the upper confidence interval of Σ4PBDE for girls incorrectly crossed the zero-line: The upper interval for girls is −0.05 and should appear below the zero-line. The corrected Figure 1 appears in this erratum.

**Figure:**
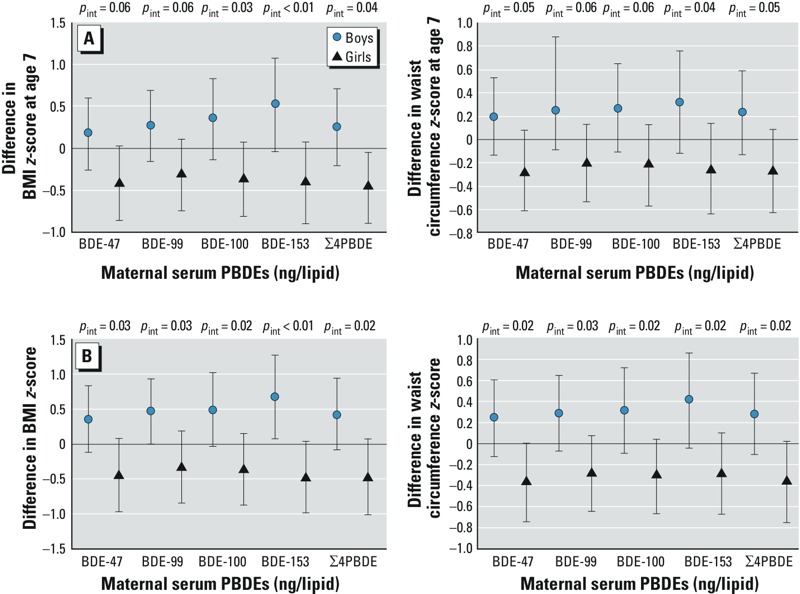
Point estimates and 95% CIs from (*A*) regression of maternal PBDE concentrations and anthropo-metric measurements at age 7 years, and (*B*) GEE model estimates of overall associations between 10-fold increases in maternal PBDE concentrations and repeated anthropometric measures (ages 2, 3.5, 5, and 7 years), with effect modification by sex, controlling for maternal age, education, prepregnancy BMI, years lived in the United States, gestational weight gain, poverty during pregnancy; and child gestational age, duration of breastfeeding, and fast food and soda consumption at age 7. *p*_int_, *p*-value for interaction.

The authors regret this error.

