# Supplementary material for: In Utero and Childhood Polybrominated Diphenyl Ether Exposures and Body Mass at Age 7 Years: The CHAMACOS Study
Source: Environ Health Perspect. 2015 Feb 27;123(6):636–42. doi: 10.1289/ehp.1408417 (PMC4455588; doi:10.1289/ehp.1408417)
Supplement: (1.8 MB) PDF [file ehp.1408417.s001.acco.pdf]

Environ Health Perspect
doi: 10.1289/ehp.1408417

Note to Readers: *EHP* strives to ensure that all journal content is accessible to all readers. However, some figures and Supplemental Material published in *EHP* articles may not conform to 508 standards due to the complexity of the information being presented. If you need assistance accessing journal content, please contact ehp508@niehs.nih.gov. Our staff will work with you to assess and meet your accessibility needs within 3 working days.

Supplemental Material

In Utero and Childhood Polybrominated Diphenyl Ether Exposures and Body Mass at Age 7 Years: The CHAMACOS Study

Ayca Erkin-Cakmak, Kim G. Harley, Jonathan Chevrier, Asa Bradman, Katherine Kogut, Karen Huen, and Brenda Eskenazi

Table S1. Summary of anthropometric measurements (mean ± SD) at each time point.

Table S2. Detection frequency and lipid-adjusted concentration (ng/g lipids) of PBDE congeners in prenatal and childhood serum.

Table S3. Correlation matrix of individual and sum of 4 PBDE congeners in maternal and child serum.

Table S4. Unadjusted and adjusted associations between 10-fold increase in *maternal* serum concentrations of PBDE (log_{10}) and child anthropometric measures at age 7.

Table S5. Adjusted associations from models with sex interaction between maternal serum concentrations of PBDE (log_{10}) and child BMI and waist circumference z-score, and obesity status at age 7.

Table S6. Adjusted associations between maternal Σ4 PBDE and BDE-153 with BMI z-score at each time point separately.

Figure S1. Directed acyclic graph Green node: Exposure variable; Dark blue node with I: Outcome variable; White node: Adjusted variable; Blue node: Ancestor of outcome; Pink node: Ancestor of both exposure and outcome variable (none shown); Green arrow: Causal pathway; Red arrow: Biasing pathway (none shown) Maternal_PBDE: Maternal PBDE levels during

pregnancy; Maternal_DM: Maternal Diabetes Mellitus; Poverty_Baseline: Poverty during pregnancy; Gestational _Age: Gestational age at birth; Child_PBDE_at_age7: Child PBDE levels at age 7.

Figure S2. Scatter plot of maternal and child Σ4PBDE concentrations and BMI z-score with regression lines at each time point (ages 2, 3.5, 5 and 7) separately for boys and girls with point estimates and 95% confidence intervals.

Table S1. Summary of anthropometric measurements (mean ± SD) at each time point.

	Age (years)	N (%)	Normal Weight (n, %)	Overweight (n, %)	Obese (n, %)	Height (cm)	Weight (kg)	BMI-z-score
**Boys**								
	2	94 (100)	69 (73.4)	10 (10.6)	15 (16.0)	86.8 ± 3.0	13.2 ± 1.9	0.5 ± 1.1
	3.5	88 (100)	41 (46.6)	23 (26.1)	24 (27.3)	99.6 ± 3.8	17.7 ± 3.6	1.2 ± 1.4
	5	91 (100)	43 (47.3)	18 (19.7)	30 (33.0)	110.2 ± 4.5	21.7 ± 5.1	1.2 ± 1.2
	7	97 (100)	48 (49.5)	19 (19.6)	30 (30.9)	123.2 ± 5.1	28.8 ± 7.8	1.1 ± 1.0
**Girls**								
	2	113 (100)	78 (69.0)	16 (14.2)	19 (16.8)	86.0 ± 3.1	13.0 ± 1.9	0.6 ± 1.2
	3.5	115 (100)	55 (47.8)	25 (21.7)	35 (30.4)	99.6 ± 3.8	17.7 ± 3.4	1.2 ± 1.1
	5	118 (100)	54 (45.8)	25 (21.2)	39 (33.0)	110.2 ± 4.3	22.1 ± 5.1	1.2 ± 1.0
	7	124 (100)	56 (45.2)	22 (17.7)	46 (37.1)	123.4 ± 5.5	29.5 ± 7.6	1.1 ± 1.0

Table S2. Detection frequency and lipid-adjusted concentration (ng/g lipids) of PBDE congeners in prenatal and childhood serum.

PBDE	Geometric Mean (95% CI)	Detection frequency (%)	Min.	10th %tile	25th %tile	50th %tile	75th %tile	90th %tile	Max
**Maternal serum PBDE levels during pregnancy (n=229)**									
BDE-47	15.32 (13.47, 17.42)	99.4	0.5	4.7	8.5	15.4	25.4	47.9	631.8
BDE-99	4.33 (3.80, 4.94)	99.4	0.3	1.5	2.3	4	6.9	12.1	260.9
BDE-100	2.77 (2.45, 3.12)	98.2	< LOD	0.9	1.6	2.5	4.3	7.7	87.7
BDE-153	2.40 (2.15, 2.69)	98.2	0.3	0.9	1.3	2.1	3.7	6.9	41.0
Σ4 PBDE	25.35 (22.42, 28.65)	100	2.3	8.4	14.1	24.3	41	73.2	1021.5
**Child serum PBDE levels at age 7 (n=216)**									
BDE-47	48.39 (43.21, 54.19)	94.2	1.9	17.4	28.4	46.4	78.3	136	768.2
BDE-99	11.32 (10.03, 12.79)	93.8	< LOD	3.8	6.6	10.7	20.4	36.9	218.2
BDE-100	10.83 (9.69, 12.10)	94.2	0.8	3.9	6.2	10.8	17.1	34.2	144.0
BDE-153	11.74 (10.57, 13.03)	94.2	0.9	4.8	6.6	11.1	19.3	32	263.1
Σ4 PBDE	83.03 (74.80, 92.17)	100	5.8	31.6	49.9	81.9	124.7	228.7	969.3

Abbreviations: Min., minimum; Max., maximum; LOD, level of detection; Σ4 PBDE, sum of BDE-47, BDE-99, BDE-100, BDE-153.

Table S3. Correlation matrix of individual and sum of 4 PBDE congeners in maternal and child serum.

PBDE	BDE-47	BDE-99	BDE-100	BDE-154	Σ4 PBDE
**Maternal serum PBDE concentrations during pregnancy**					
BDE-47	1				
BDE-99	0.92*	1			
BDE-100	0.94*	0.90*	1		
BDE-154	0.77*	0.72*	0.89*	1	
Σ4 PBDE	0.99*	0.95*	0.97*	0.84*	1
**Child serum PBDE concentrations during pregnancy**					
BDE-47	1				
BDE-99	0.97*	1			
BDE-100	0.95*	0.94*	1		
BDE-154	0.65*	0.67*	0.76*	1	
Σ4 PBDE	0.98*	0.97*	0.97*	0.77*	1
**Maternal serum PBDE concentrations during pregnancy and child serum PBDE concentration at age 7**					
BDE-47	0.27*	0.23*	0.25*	0.21	0.26*
BDE-99	0.25*	0.22	0.23*	0.18	0.24*
BDE-100	0.27*	0.25*	0.26*	0.24*	0.27*
BDE-153	0.28*	0.24*	0.26*	0.29*	0.28*
Σ4 PBDE	0.27*	0.23*	0.26*	0.23*	0.27*

Abbreviations: Σ4 PBDE, sum of BDE-47, BDE-99, BDE-100, BDE-153.

*p <0.05 statistically significant.

Table S4. Unadjusted and adjusted associations between 10-fold increase in *maternal* serum concentrations of PBDE (log_{10}) and child anthropometric measures at age 7.

PBDE	BMI z-score β (95% CI)	Waist Circumference z-score β (95% CI)	Overweight OR (95% CI)	Obese OR (95% CI)
**Crude**				
BDE-47	-0.001 (-031, 0.31)	1.85 (-1.13, 4.84)	0.97 (0.52, 1.79)	1.15 (0.60, 2.18)
BDE-99	0.08 (-0.23, 0.39)	2.32 (-0.62, 5.25)	1.04 (0.57, 1.90)	1.29 (0.69, 2.43)
BDE-100	0.02 (-0.31, 0.35)	1.44 (-1.73, 4.60)	0.98 (0.51, 1.89)	1.17 (0.59, 2.31)
BDE-153	-0.07 (-0.44, 0.29)	0.23 (-3.25, 3.71)	0.87 (0.43, 1.78)	1.06 (0.50, 2.25)
Σ4 PBDE	0.01 (-0.33, 0.33)	1.81 (-1.35, 4.99)	0.95 (0.49, 1.82)	1.16 (0.59, 2.30)
**Adjusted**				
BDE-47	-0.10 (-0.41, 0.21)	0.01 (-0.24, 0.26)	0.79 (0.38, 1.67)	1.03 (0.48, 2.23)
BDE-99	-0.01 (-0.31, 0.29)	0.04 (-0.19, 0.27)	0.87 (0.43, 1.76)	1.12 (0.54, 2.32)
BDE-100	-0.03 (-0.37, 0.30)	0.03 (-0.24, 0.30)	0.89 (0.40, 1.97)	1.19 (0.52, 2.73)
BDE-153	-0.002 (-0.38, 0.30)	0.03 (-0.28, 0.34)	1.05 (0.42, 2.63)	1.59 (0.61, 4.16)
Σ4 PBDE	-0.08 (-0.41, 0.25)	0.02 (-2.45, 0.28)	0.82 (0.38, 1.79)	1.09 (0.49, 2.46)

Abbreviations: BMI, body mass index; Σ4 PBDE, sum of BDE-47, BDE-99, BDE-100, BDE-153.

Overweight: Age- and sex-specific BMI ≥ the 85th percentile; Obese: Age- and sex-specific BMI ≥ the 95th percentile.

Adjusted model includes maternal age, education, pre-pregnancy BMI, years lived in US, gestational weight gain, poverty during pregnancy; and child gestational age, duration of breast feeding, and fast food and soda consumption at age 7.

Table S5. Adjusted associations from models with sex interaction between maternal serum concentrations of PBDE ($\log_{10}$) and child BMI and waist circumference z-score, and obesity status at age 7.

PBDE	BMI z-score β (95% CI)	Interaction *p*-value	Waist circumference z-score β (95% CI)	Interaction *p*-value	Overweight OR (95% CI)	Interaction *p*-value	Obese OR (95% CI)	Interaction *p*-value
**BDE-47**								
Boys	0.19 (-0.24, 0.61)		0.20 (-0.13, 0.53)		1.27 (0.46, 3.50)		2.82 (0.87, 9.03)	
Girls	-0.40 (-0.84, 0.04)	0.06	-0.26 (-0.60, 0.08)	0.05	0.46 (0.16, 1.38)	0.18	0.37 (0.11, 1.20)	0.02
**BDE-99**								
Boys	0.28 (-0.14, 0.70)		0.25 (-0.08, 0.87)		1.34 (0.49, 3.66)		3.18 (1.04, 9.77)	
Girls	-0.29 (-0.72, 0.12)	0.05	-0.19 (-0.52, 0.13)	0.06	0.56 (0.20, 1.54)	0.23	4.23 (0.14, 1.28)	0.01
**BDE-100**								
Boys	0.36 (-0.12, 0.84)		0.27 (-0.10, 0.65)		1.57 (0.49, 4.95)		4.32 (1.17, 15.9)	
Girls	-0.35 (-0.79, 0.09)	0.03	-0.21 (-0.56, 0.13)	0.06	0.53 (0.18, 1.57)	0.17	0.42 (0.13, 1.34)	<0.01
**BDE-153**								
Boys	0.54 (-0.02, 1.08)		0.32 (-0.11, 0.76)		2.29 (0.60, 8.74)		10.5 (2.19, 49.8)	
Girls	-0.39 (-0.88, 0.09)	0.01	-0.21 (-0.56, 0.13)	0.04	0.56 (0.17, 1.86)	0.11	0.41 (0.12, 1.53)	<0.01
**Σ4 PBDE**								
Boys	0.26 (-0.19, 0.72)		0.24 (-0.12, 0.59)		1.34 (0.45, 3.98)		3.45 (1.01, 11.8)	
Girls	-0.41 (-0.87, -0.05)	0.04	-0.26 (-0.62, 0.09)	0.05	0.48 (0.16, 1.47)	0.19	0.36 (0.10, 1.23)	0.01

Abbreviations: BMI, body mass index; OR, odds ratio; Σ4 PBDE, sum of BDE-47, BDE-99, BDE-100, BDE-153.

Overweight: Age- and sex-specific BMI ≥ the 85th percentile; Obese: Age- and sex-specific BMI ≥ the 95th percentile.

Controlling for maternal age, education, pre-pregnancy BMI, years lived in US, gestational weight gain, poverty during pregnancy; and child gestational age, duration of breast feeding, and fast food and soda consumption at age 7.

Table S6. Adjusted associations between maternal Σ4 PBDE and BDE-153 with BMI z-score at each time point separately.

Age (years)	BMI z-score β (95% CI) Boys	BMI z-score β (95% CI) Girls	Interaction *p*-value
**Maternal serum Σ4 PBDE**			
2	0.38 (-0.15, 0.91)	-0.56 (-1.11, -0.02)*	0.01
3.5	0.62 (0.07, 1.18)*	-0.66 (-1.19, -0.11)*	<0.01
5	0.34 (-0.16, 0.85)	-0.42 (-0.92, 0.09)	0.03
7	0.26 (-0.19, 0.71)	-0.41 (-0.87, 0.04)	0.04
**Maternal serum PBDE-153**			
2	0.39 (-0.25, 1.02)	-0.66 (-1.24, -0.09)*	0.01
3.5	0.99 (0.32, 1.66)*	-0.64 (-1.23, -0.06)*	<0.01
5	0.58 (-0.03, 1.19)**	-0.39 (-0.92, 0.14)	0.01
7	0.54 (-0.02, 1.08)**	-0.39 (-0.88, 0.09)	<0.01

Abbreviations: BMI, body mass index; Σ4 PBDE, sum of BDE-47, BDE-99, BDE-100, BDE-153.

Controlling for maternal age, education, pre-pregnancy BMI, years lived in US, gestational weight gain, poverty during pregnancy; and child gestational age, duration of breast feeding, and fast food and soda consumption at each time point (age 2, 3.5, 5 and 7 years) separately.

$^{*}p < 0.05$, $^{**}p < 0.1$.

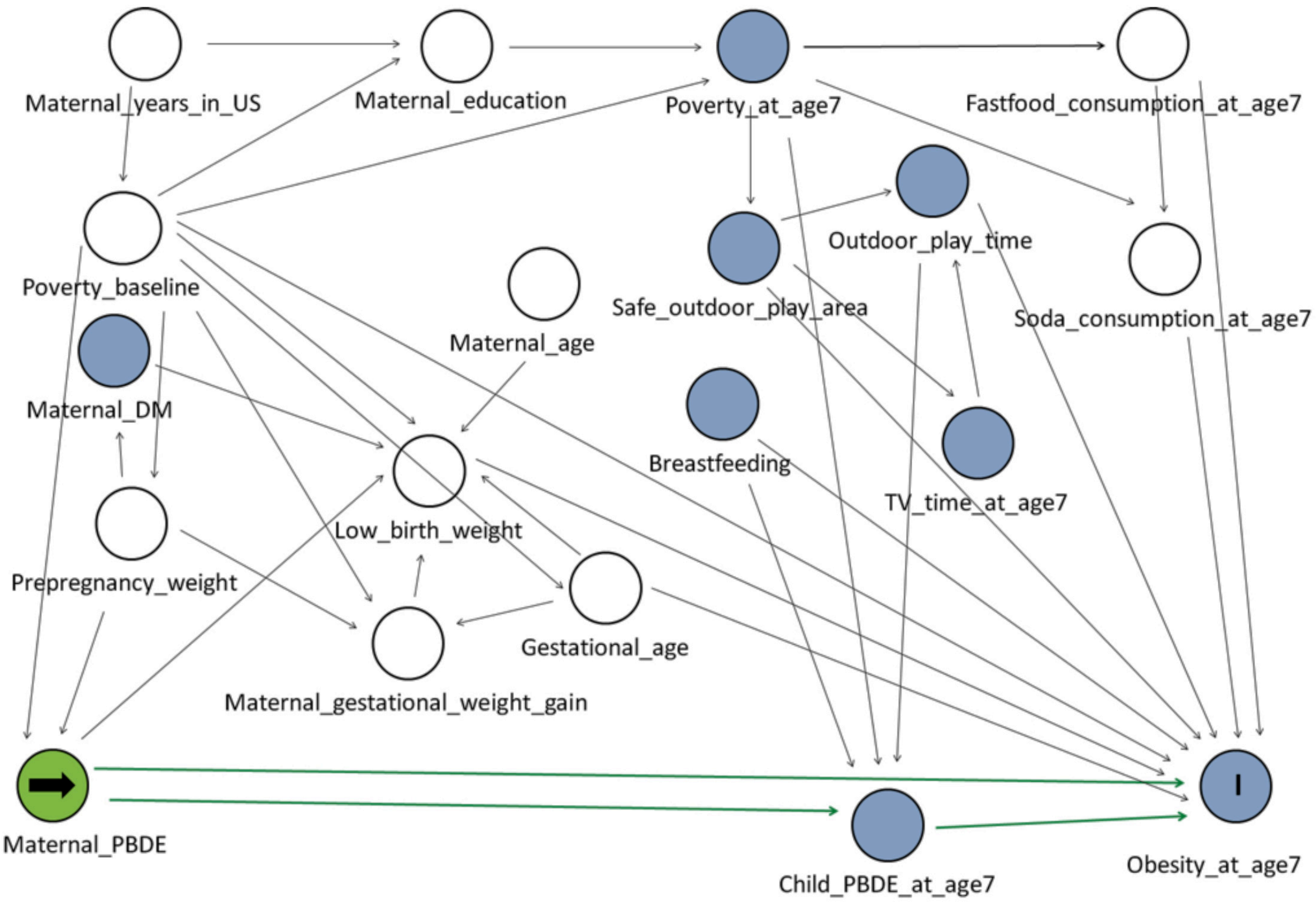

Figure S1. Directed acyclic graph Green node: Exposure variable; Dark blue node with I: Outcome variable; White node: Adjusted variable; Blue node: Ancestor of outcome; Pink node: Ancestor of both exposure and outcome variable (none shown); Green arrow: Causal pathway; Red arrow: Biasing pathway (none shown) Maternal_PBDE: Maternal PBDE levels during pregnancy; Maternal_DM: Maternal Diabetes Mellitus; Poverty_Baseline: Poverty during pregnancy; Gestational _Age: Gestational age at birth; Child_PBDE_at_age7: Child PBDE levels at age 7.

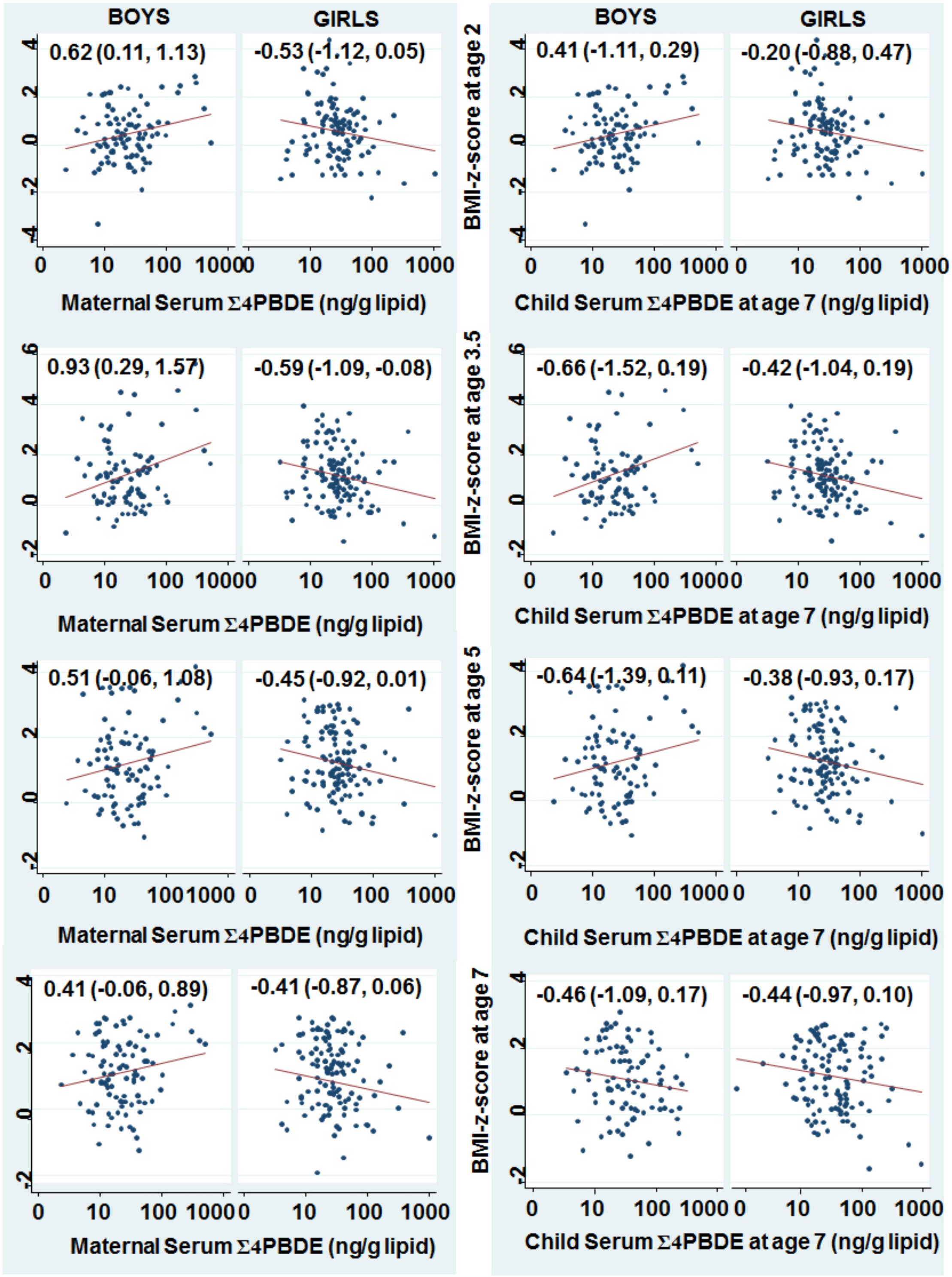

Figure S2. Scatter plot of maternal and child Σ4PBDE concentrations and BMI z-score with regression lines at each time point (ages 2, 3.5, 5 and 7) separately for boys and girls with point estimates and 95% confidence intervals.
